# Sympathoexcitatory Responses to Isometric Handgrip Exercise Are Associated With White Matter Hyperintensities in Middle-Aged and Older Adults

**DOI:** 10.3389/fnagi.2022.888470

**Published:** 2022-07-11

**Authors:** Andrew G. Pearson, Kathleen B. Miller, Adam T. Corkery, Nicole A. Eisenmann, Anna J. Howery, Karly A. Cody, Nathaniel A. Chin, Sterling C. Johnson, Jill N. Barnes

**Affiliations:** ^1^Bruno Balke Biodynamics Laboratory, Department of Kinesiology, University of Wisconsin-Madison, Madison, WI, United States; ^2^Waisman Laboratory for Brain Imaging and Behavior, University of Wisconsin-Madison School of Medicine and Public Health, Madison, WI, United States; ^3^Division of Geriatrics and Gerontology, Department of Medicine, University of Wisconsin-Madison School of Medicine and Public Health, Madison, WI, United States; ^4^Alzheimer’s Disease Research Center, University of Wisconsin-Madison School of Medicine and Public Health, Madison, WI, United States; ^5^Geriatric Research Education and Clinical Center, William S. Middleton Hospital Department of Veterans Affairs, Madison, WI, United States

**Keywords:** blood pressure, cerebrovascular disease, white matter, cerebral artery blood velocity, cerebrovascular resistance

## Abstract

Vascular dysfunction may occur prior to declines in cognitive function and accumulation of neuropathology. White matter hyperintensities (WMH) develop due to cerebral ischemia and elevated blood pressure in midlife. The purpose of this study was to evaluate associations between cardiovascular and cerebrovascular responses to sympathoexcitatory stimuli and WMH burden in cognitively unimpaired middle-aged and older adults. Sixty-eight adults (age = 63 ± 4y, men = 20, women = 48) participated in this study. Participants completed isometric handgrip exercise (IHG) exercise at 40% of maximal voluntary contraction until fatigue followed by a 90s period of post-exercise ischemia. Heart rate (HR), mean arterial pressure (MAP), middle cerebral artery blood velocity (MCAv), and end-tidal CO_2_ were continuously measured throughout the protocol. Cerebrovascular resistance index (CVRi) was calculated as MAP/MCAv. WMH lesion volume and intracranial volume (ICV) were measured using a FLAIR and T1 scan on a 3T MRI scanner, respectively. WMH fraction was calculated as (WMH lesion volume/ICV)*100 and cubic root transformed. Multiple linear regressions were used to determine the association between cardiovascular and cerebrovascular responses to IHG exercise and post-exercise ischemia and WMH fraction. Multiple linear regression models were adjusted for age, sex, apolipoprotein ε4 status, and total work performed during IHG exercise. During IHG exercise, there were significant increases from baseline in HR (25 ± 12%), MAP (27 ± 11%), MCAv (5 ± 10%), and CVRi (22 ± 17%; *P* < 0.001 for all). During post-exercise ischemia, HR (8 ± 7%), MAP (22 ± 9%), and CVRi (23 ± 16%) remained elevated (*P* < 0.001) while MCAv (0 ± 10%) was not different compared to baseline. There was an inverse association between the percent change in HR (r = −0.42, *P* = 0.002), MAP (r = −0.41, *P* = 0.002), and CVRi (r = −0.31, *P* = 0.045), but not MCAv (r = 0.19, *P* = 0.971) in response to IHG exercise and WMH fraction. There were no associations between responses to post-exercise ischemia and WMH fraction. Lower sympathoexcitatory responses to IHG exercise are associated with greater WMH burden in middle-aged to older adults. These findings suggest that individuals who demonstrate smaller increases in HR, MAP, and CVRi in response to sympathoexcitatory stress have greater WMH burden.

## Introduction

Identification of novel vascular risk factors is necessary to understand vascular contributions to the progression of cognitive decline and dementia (Gorelick et al., 2011). Specifically, investigating mechanisms underlying the reduction in cerebral blood flow and changes in blood pressure (BP) during the presymptomatic phase of dementia could help to elucidate the link between vascular risk and dementia (Snyder et al., 2015). Impaired peripheral and cerebral vascular regulation as a consequence of chronic hypertension could contribute to the reduction in cerebral blood flow and progression of cognitive decline (Farkas and Luiten, 2001). Indeed, vascular dysregulation and impaired hemodynamic responses to stress may occur early in the progression of cognitive decline, prior to clinically-relevant declines in cognitive function (Iadecola, 2004; Iturria-Medina et al., 2016). In fact, vascular risk scores have been used to predict future risk of dementia 20 years later in middle-aged adults (Kivipelto et al., 2006). Further, in individuals with Alzheimer’s disease (AD), vascular factors including systolic hypertension and angina at the time of AD diagnosis are associated with a more rapid decline in cognitive function (Mielke et al., 2007).

White matter hyperintensities (WMH) are areas of increased brightness and signal density observed on MRI scans indicative of small vessel cerebrovascular disease which are associated with cognitive decline and increased severity of dementia in individuals with AD (Heyman et al., 1998). Longitudinal studies suggest an association between WMH burden and cardiovascular risk factors (Breteler et al., 1994; Jeerakathil et al., 2004), lower total cerebral perfusion (Vernooij et al., 2008), and lower cerebral blood flow (Promjunyakul et al., 2018). Indeed, cerebral blood flow is lower in areas with WMH compared to areas without WMH (Brickman et al., 2009; Stewart et al., 2021). Cerebral blood flow is also lower in adults with WMH compared to those without (Fazekas et al., 1988; O’Sullivan et al., 2002). In addition, lower middle cerebral artery blood velocity (MCAv) and higher cerebral pulsatility index (PI) has been reported in adults with greater severity of WMH burden (Puglisi et al., 2018). Importantly, during the presymptomatic phase of dementia, WMH volumes are associated with increased risk of progression to MCI (Soldan et al., 2020).

Longitudinal studies have demonstrated that elevated BP and systolic blood pressure (SBP) variability in midlife increases the risk of developing WMH (Dufouil et al., 2001; Havlik et al., 2002; Wartolowska and Webb, 2021). In addition to vascular risk factors, the physiological response to repeated acute or chronic physiological and psychological stressors likely contribute to future risk of disease (McEwen and Stellar, 1993). The response to acute physiological stressors may therefore reveal dysfunction in the systemic and cerebral circulation that could affect white matter health. For example, a greater increase in BP in response to mental stress is associated with WMH burden (Waldstein et al., 2004) and impaired hypercapnic cerebrovascular reactivity is associated with white matter damage (Sam et al., 2016). Isometric handgrip (IHG) exercise represents an acute sympathoexcitatory stressor indicative of activities of daily living as heart rate (HR), BP, and sympathetic nerve activity increase in response to sustained isometric contractions (Lind and McNicol, 1967; Mark et al., 1985). Post-exercise ischemia occludes blood flow to the previously contracted muscle leading to sustained elevations in BP and sympathetic activity, in the absence of muscle contraction (Rowell and O’Leary, 1990). Together, IHG exercise and post-exercise ischemia provide insight into the cardiovascular responses to muscle mechanoreflex and metaboreflex activation. Isometric and rhythmic handgrip exercise responses have been used to evaluate cardiovascular risk in a variety of populations (Ide et al., 1998; Giller et al., 2000; Ranadive et al., 2017; Miller et al., 2019; Tarumi et al., 2021). We have previously reported that women with a history of preeclampsia (who have an elevated risk of cerebrovascular disease and cognitive decline) demonstrated a greater cerebral blood flow velocity response to IHG exercise (Miller et al., 2019), despite similar increases in BP (Ranadive et al., 2017), compared with women without a history of preeclampsia. However, the cardiovascular and cerebrovascular response to IHG exercise and post-exercise ischemia have not been linked to white matter health. The purpose of this study was to evaluate associations between cardiovascular and cerebrovascular responses to sympathoexcitatory stimuli and WMH burden in a cohort of cognitively unimpaired middle-aged and older adults. We hypothesized that both a greater cardiovascular and cerebrovascular response to IHG exercise and post-exercise ischemia would be associated with greater WMH burden.

## Materials and Methods

### Ethics Statement

All study procedures were approved by the University of Wisconsin-Madison Institutional Review Board and performed according to the Declaration of Helsinki by obtaining written informed consent from each participant.

### Participants

Participants were recruited from cohorts within the Wisconsin Alzheimer’s Disease Research Center (ADRC). These cohorts included the Investigating Memory in Preclinical Alzheimer’s Disease – Causes and Treatments (IMPACT) cohort and the Healthy Older Controls cohort. Within the IMPACT cohort, participants had either 1) a parent clinically diagnosed with AD clinical syndrome according to the self-report of the adult child, 2) a biological mother or father that lived to at least 75 years old or 70 years old, respectively, without symptoms of dementia, or 3) indeterminate parental history of dementia (i.e., parent did not live to age limits described above, parent has or had MCI, or participant is adopted and biological family history is unknown, etc.). Within the Healthy Older Control cohort, participants were at least 65 years old and did not have a family history of dementia. Family history of dementia was determined using a self-report dementia questionnaire for each parent or an autopsy report when available (Johnson et al., 2017). Participants were considered to be cognitively unimpaired based on a consensus diagnosis as previously described (Sager et al., 2005; Rivera-Rivera et al., 2016). Briefly, at least one physician and at least one neuropsychologist met to determine the stage of dementia severity using the Clinical Dementia Rating scale (Morris, 1997). Additionally, participants underwent a blood draw and MRI (Rivera-Rivera et al., 2016).

Ninety-five cognitively unimpaired adults (32 men, 63 women) between 55 and 69 years of age participated in this study. The distribution of men and women in this study is similar to a previously published study using the Wisconsin ADRC IMPACT cohort (Hoscheidt et al., 2016). All participants had a body mass index (BMI) less than or equal to 34.9 kg/m^2^. Women were postmenopausal for >1 year and were not currently taking oral menopausal hormone therapy. Exclusion criteria consisted of a confirmed diagnosis of MCI or dementia of any kind, uncontrolled hypertension, significant surgical history, history of clinically significant stroke, cerebrovascular disease, or other major neurological disorders. Participants with controlled hypertension were included in the study.

### Experimental Procedures

Participants visited the Bruno Balke Biodynamics Laboratory at the University of Wisconsin-Madison on two separate occasions for a screen day and experimental study day. On the experimental study day, participants arrived at the laboratory after a 4-h fast and having refrained from performing strenuous exercise in the previous 24 h. Participants were instructed to avoid consumption of caffeine or chocolate in the previous 12 h, alcohol in the previous 24 h, and avoid the use of aspirin or non-steroidal anti-inflammatory drugs for 48 h prior to the study visit. Participants were asked to withhold over-the-counter medications on the experimental study day. All experimental study day procedures were performed while participants lied supine in a dimly lit, temperature-controlled room kept between 22 and 24°C. On a separate day, participants visited the Wisconsin Institutes for Medical Research in Madison, WI for an MRI scan.

### Screen Day Measurements

Upon arrival to the laboratory, height and weight were obtained using a standard scale and stadiometer. The screen day visit consisted of a brief familiarization with study procedures, a supine arterial BP measurement using a brachial cuff (Datex Ohmeda, GE Healthcare, Fairfield, CT, United States) after resting quietly in a dimly lit room for 10 min, and middle cerebral artery (MCA) screening using transcranial Doppler ultrasound (TCD). MCA imaging was conducted to ensure proper angle of the probe, and the anatomical location, depth of the signal, and mean velocity were recorded. Maximal voluntary contraction (MVC) was determined from the average of two brief maximal handgrip contractions of the left hand separated by 1 min of rest.

### Experimental Study Day Measurements

Following 10 min of supine rest, arterial BP was measured in triplicate using a brachial BP cuff and the average value was recorded. HR was measured using a 3-lead electrocardiogram (CardioCap, Datex Ohmeda, GE Healthcare, Fairfield, CT, United States). Mean arterial blood pressure (MAP), SBP, and diastolic blood pressure (DBP) were continuously measured using a non-invasive finger cuff (NOVA, Finapres Medical Systems, Amsterdam, The Netherlands). End-tidal CO_2_ (ETCO_2_) was measured using a nasal cannula. MCAv of the left MCA was measured using a 2-MHz TCD (Spencer Technologies, Seattle, WA, United States). The ipsilateral MCA was used to measured MCAv to represent global changes in cerebral blood flow. Quality MCAv signals were obtained using established guidelines (DeWitt and Wechsler, 1988) and signal quality was confirmed prior to beginning the IHG exercise protocol.

### Experimental Study Day Protocol

**FIGURE 1:**
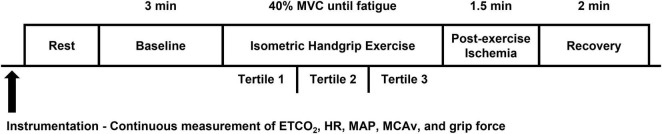
Experimental study day timeline. ETCO_2_, end-tidal CO_2_; HR, heart rate; MAP, mean arterial pressure; MCAv, middle cerebral artery blood velocity.

Participants were fitted with a BP cuff on the upper left arm (D. E. Hokanson, Inc., Bellevue, WA) and a handgrip dynamometer (AD Instruments, Colorado Springs, United States) was placed in their left hand. Due to the experimental setup, all participants performed the IHG exercise protocol using their left hand apart from two participants due to medical history. Prior to exercise, participants rested quietly in the supine position for 3 min while baseline measurements were collected. Then, participants completed an IHG exercise protocol at 40% of their previously determined MVC until fatigue as previously described (Ranadive et al., 2017; Miller et al., 2019). Fatigue was defined as the time at which participants were no longer able to maintain 40 ± 5% of their MVC despite verbal encouragement by the researchers. Upon reaching fatigue, a BP cuff was rapidly inflated to a pressure 100 mmHg above resting SBP to elicit a period of post-exercise ischemia. Following 90 s of cuff inflation, participants rested quietly for 2 min. All cardiovascular and cerebrovascular variables were closely monitored throughout the experimental protocol. The percentage of MVC was continuously monitored throughout the protocol and participants were verbally instructed and encouraged to maintain the target intensity. Grip force (kg) was recorded throughout the protocol. An experimental study day timeline is shown in Figure 1.

### MRI Measurements

On a separate visit, MRI brain scans were done on a 3T clinical MRI scanner (MR750, GE Healthcare, Waukesha, WI, United States) at the Wisconsin Institutes for Medical Research. In the supine position, participants were fitted and imaged with a 32-channel or 48-channel head coil (Nova Medical Head Coil, Nova Medical, Wilmington, MA, United States) with a gradient strength of 50 mT/m and a gradient slew rate of 200 mT/m/ms. Intracranial volume (ICV) was measured using a T1-weighted structural brain volume (BRAVO) scan with the following parameters: fast spoiled gradient echo sequence, inversion time = 450 ms, repetition time = 8.1 ms, echo time = 3.2 ms, flip angle = 12°, acquisition matrix = 256 × 256, field of view = 256 mm, slice thickness = 1.0 mm, and scan time ∼8 min. Total WMH lesion volume was measured using a FLAIR scan.

### Apolipoprotein E Genotyping

Apolipoprotein E (*APOE*) is a genetic risk factor for Alzheimer’s disease and presence of one or more copies of the ε4 allele is associated with increased risk of cognitive decline (Liu et al., 2013). *APOE* status was determined using competitive allele-specific polymerase chain reaction-based genotyping assays (LGC Genomics, Beverly, MA) as previously described (Johnson et al., 2017). Participants were considered *APOE* ε4 positive if they had one or more copies of the ε4 allele.

### Data Analysis

Cardiovascular and cerebrovascular variables were recorded using LabChart 8 at 250 Hz (AD Instruments, Dunedin, New Zealand) and stored offline for analysis. Cardiovascular variables included: HR, cardiac output (CO), MAP, SBP, DBP, and pulse pressure (PP). Cerebrovascular variables included: MCAv, cerebral PI, and cerebrovascular resistance index (CVRi). CO was calculated as HR x stroke volume. Stroke volume was derived from finger BP using Modelflow analysis (NOVA, Finapres Medical Systems). Cerebral PI was calculated as (systolic MCAv – diastolic MCAv)/mean MCAv. CVRi was calculated as MAP/MCAv. ETCO_2_ was measured throughout the protocol. Baseline measurements were recorded during the middle 60 s of the 3 min baseline period prior to exercise. Variables were continuously recorded during the IHG exercise and post-exercise ischemia protocols. Time to fatigue was recorded for each participant in seconds. Due to individual differences in handgrip MVC and time to fatigue, work performed was derived by calculating the integral of grip force throughout the IHG exercise protocol. To account for individual differences in the time to fatigue during IHG exercise and consistent with our previous work, data was divided into tertiles for analysis (Ranadive et al., 2017; Miller et al., 2019). Briefly, the time to fatigue for each participant was recorded during the IHG exercise protocol using an electronic handgrip device interfaced with LabChart. Then, the response to IHG exercise was divided into tertiles for analysis. Cardiovascular and cerebrovascular variables were averaged during each tertile of IHG exercise. The response to post-exercise ischemia during the final 60 s was used for analysis. The percent change from baseline during the IHG exercise and post-exercise ischemia protocol was calculated for all cardiovascular and cerebrovascular variables and the average of each variable of interest was used for analysis.

WMH were assessed using a lesion prediction algorithm from the Lesion Segmentation Tool (LST) in SPM (Schmidt, 2017). The LST lesion prediction algorithm uses a FLAIR scan (with the optional co-registration and resampling to the resolution of a T1-weighted reference image) to estimate the lesion probability at each voxel, outputting a lesion probability map. In the quantification step, the lesion probability was thresholded to 0.5 and constrained to voxels at least 4mm from the estimated edge of brain tissue. The resulting lesion probability map for each scan underwent visual quality assessment by trained reviewers, where the probability map was overlayed onto the FLAIR scan and compared side by side for accuracy to the unsegmented FLAIR scan. As needed, segmentations were secondarily reviewed by an experienced neuroradiologist and excluded when warranted. WMH fraction was calculated by dividing WMH lesion volume (mm^3^) by ICV (mm^3^) and multiplying by 100. As WMH lesion volume and fraction are highly skewed measures, data were cubic root transformed for analysis to produce a normal distribution and reduce skewness (Habes et al., 2016).

### Statistical Analysis

Normality of all variables was assessed using Shapiro-Wilk tests and visually inspected using histograms and QQ plots. Equal variance across time points (baseline, each tertile of IHG exercise, and post-exercise ischemia) for each variable was assessed using Brown-Forsythe tests. One-way repeated measures ANOVA were performed to evaluate if raw cardiovascular and cerebrovascular variables differed from baseline during the IHG exercise and post-exercise ischemia protocol. One-way repeated measures ANOVA were performed to evaluate if the percent change from baseline in cardiovascular and cerebrovascular variables differed between time points (each tertile of IHG exercise and post-exercise ischemia). Pairwise comparisons for each variable were assessed using Bonferroni *post hoc* testing and effect sizes were calculated as eta squared. All statistical analyses were completed using R software. Means ± standard deviation for all variables are presented. Statistical significance was set *a priori* at *P* < 0.05.

**FIGURE 2:**
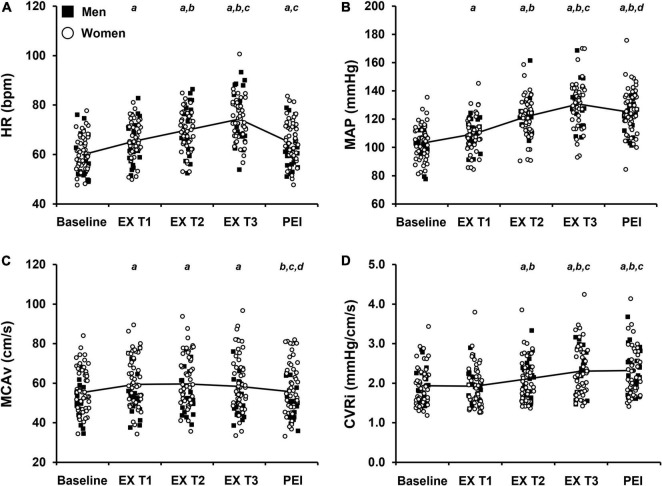
Raw values of cardiovascular and cerebrovascular variables at baseline and during each tertile of isometric handgrip exercise (EX T1, EX T2, EX T3, respectively) followed by a period of post-exercise ischemia (PEI). Means and individual data presented. The solid black line represents mean values. **(A)** Heart rate, HR; **(B)** Mean arterial pressure, MAP; **(C)** Middle cerebral artery blood velocity, MCAv; **(D)** Cerebrovascular resistance index, CVRi. Significance is indicated by the following: *^a^P* < 0.05 vs. baseline, *^b^P* < 0.05 vs. exercise T1, *^c^P* < 0.05 vs. exercise T2, *^d^P* < 0.05 vs. exercise T3 (n = 68; One-way repeated measures ANOVA with Bonferroni *post hoc* testing).

**FIGURE 3:**
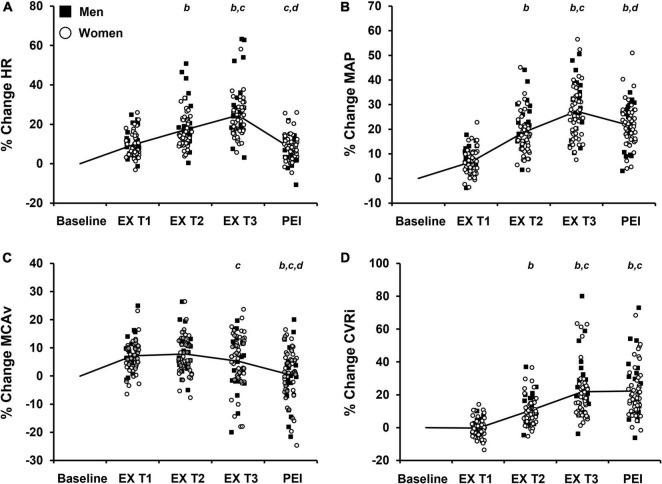
Percent change (% change) from baseline values in cardiovascular and cerebrovascular variables during each tertile of isometric handgrip exercise (EX T1, EX T2, EX T3, respectively) followed by a period of post-exercise ischemia (PEI). Means and individual data presented. The solid black line represents mean values. **(A)** % change in heart rate, HR; **(B)** % change in mean arterial pressure, MAP; **(C)** % change in middle cerebral artery blood velocity, MCAv; **(D)** % change in cerebrovascular resistance index, CVRi. Significance is indicated by the following: *^b^P* < 0.05 vs. exercise T1, *^c^P* < 0.05 vs. exercise T2, *^d^P* < 0.05 vs. exercise T3 (n = 68; One-way repeated measures ANOVA with Bonferroni *post hoc* testing).

To determine the association between the cardiovascular and cerebrovascular response to IHG exercise and post-exercise ischemia and WMH fraction, multiple linear regression was performed. The final tertile of IHG exercise was used for statistical analysis as the final tertile represents the greatest sympathoexcitatory stress experienced by participants prior to onset of fatigue as indicated by a progressive increase in HR and MAP in all participants (Figures 2, 3 and Ranadive et al., 2017). In each multiple linear regression analysis, independent variables of age at MRI, sex, *APOE* ε4 status, and work performed during the IHG exercise protocol were added to the model as independent variables. Age at MRI was included as an independent variable in the model due to increases in WMH burden with age (de Leeuw et al., 2001). For sex, women were assigned a value of 1 and men assigned a value of 0 as WMH burden is greater in women compared to men (Fatemi et al., 2018). WMH burden is associated with *APOE* status such that homozygous *APOE* ε4 individuals have greater rates of WMH accumulation compared to individuals without a copy of the *APOE* ε4 allele (Sudre et al., 2017). Accordingly, participants with one or more copies of the *APOE* ε4 allele were considered *APOE* ε4 positive and assigned a value of 1 while participants without a copy were considered *APOE* ε4 negative and assigned a value of 0 (Kaufman et al., 2021). Finally, the pressor response to handgrip exercise may be dependent upon absolute contraction loads during exercise (Lee et al., 2021). Therefore, work performed during the IHG exercise protocol was added as an independent variable to account for inter-individual differences in MVC and absolute contraction load during exercise.

Participants with controlled hypertension were included in the study. As such, we also evaluated the effect of controlled hypertension on our results. To determine the influence of controlled hypertension on our results, participants with controlled hypertension (*n* = 13) were assigned a value of 1 while normotensive participants (*n* = 55) were assigned a value of 0 (Dufouil et al., 2001) when added to the multiple linear regression models.

Due to the uneven distribution of men and women, we did not evaluate sex differences in the cardiovascular and cerebrovascular response to IHG exercise and post-exercise ischemia or associations with WMH fraction *a priori*.

## Results

### Participants

Of the 95 participants recruited for this study, 27 participants were not included in the final analysis due to not completing the IHG exercise protocol (*n* = 2), inadequate data quality (i.e., MAP or MCAv signal loss; *n* = 8), incomplete *APOE* genotype data (*n* = 7), or incomplete WMH data (*n* = 9). In addition, one participant was excluded from analysis due to having a WMH lesion volume > 7 standard deviations above the mean (mean WMH lesion volume = 776.0 mm^3^). Participant characteristics and cardiovascular variables at rest in the 68 participants (20 men, 48 women) with complete data are located in Table 1. The average age of participants during their most recent MRI was 62 ± 4 years. The average difference between the ages of participants at their most recent MRI to the experimental study day visit in the laboratory was 0.2 years. The average WMH lesion volume was 776.0 ± 1118.4 mm^3^, average ICV was 1.4 × 10^6^ ± 1.4 × 10^5^ mm^3^, and average WMH fraction was 0.05 ± 0.07. Sixty-two participants (91%) were right hand dominant.

**TABLE 1:** Participant characteristics and selected cardiovascular variables at rest.

Variable	Value
Sex (M/W)	20/48
Age (y)	63 ± 4
Education (y)	17 ± 3
Weight (kg)	76 ± 15
Height (cm)	168 ± 8
Body mass index (kg/m^2^)	27 ± 4
Heart rate (bpm)	60 ± 7
Mean arterial blood pressure (mmHg)	93 ± 10
Systolic blood pressure (mmHg)	126 ± 15
Diastolic blood pressure (mmHg)	76 ± 8
MoCA	28 ± 2
Family history positive (n,%)	48, 71
*APOE* ε4 positive (n,%)	27, 40
Hypertension (n,%)	13, 19
Diabetes (n,%)	2, 3

*Values expressed as mean ± SD. MoCA, Montreal Cognitive Assessment; APOE, apolipoprotein E.*

### Cardiovascular and Cerebrovascular Responses to Sympathoexcitatory Stimuli

The average handgrip MVC for participants was 25.3 ± 10.0 kg. The average time to fatigue during IHG exercise was 153.6 ± 49.1 s and average work performed during IHG exercise was 1,371.4 ± 539.8 kg/s. During IHG exercise, HR, CO, MAP, PP, MCAv, and CVRi increased compared with baseline (*P* < 0.001; Figure 2 and Table 2), as expected. Cerebral PI decreased during IHG exercise compared with baseline (*P* < 0.001; Table 2). During post-exercise ischemia, HR, CO, MAP, PP, and CVRi remained elevated compared with baseline (*P* < 0.001) whereas MCAv did not differ compared with baseline values (*P* > 0.05; Figure 2 and Table 2). Cerebral PI increased during post-exercise ischemia compared with baseline (*P* < 0.001, Table 2). ETCO_2_ was similar between baseline and IHG exercise until the final tertile of IHG exercise when ETCO_2_ decreased and remained lower during post-exercise ischemia (*P* < 0.001, Table 2). The percent change from baseline in HR, MAP, MCAv, and CVRi during IHG exercise and post-exercise ischemia are presented in Figure 3. The percent change from baseline in all cardiovascular and cerebrovascular variables during IHG exercise and post-exercise ischemia are located in Table 3.

**TABLE 2:** Cardiovascular and cerebrovascular variables during isometric handgrip exercise and post-exercise ischemia.

Variable	Baseline	Exercise T1	Exercise T2	Exercise T3	PEI	*P*-value (effect size)
HR (bpm)	60 ± 7	65±8^*a*^	70 ± 8^*a,b*^	74 ± 9^*a,b,c*^	65 ± 8^*a,c,d*^	**<0.001** (0.266)
CO (L/min)	5.31.2	5.3 ± 1.2	5.6 ± 1.3^*a,b*^	6.0 ± 1.4^*a,b,c*^	5.5 ± 1.2^*a,b,d*^	**<0.001** (0.045)
MAP (mmHg)	10312	110±12^*a*^	122 ± 14^*a,b*^	131 ± 16^*a,b,c*^	125 ± 15^*a,b,d*^	**<0.001** (0.359)
SBP (mmHg)	14318	150±19^*a*^	166 ± 21^*a,b*^	178 ± 23^*a,b,c*^	175 ± 23^*a,b,c*^	**<0.001** (0.310)
DBP (mmHg)	76 ± 9	81±9^*a*^	89 ± 11^*a,b*^	95 ± 13^*a,b,c*^	89 ± 10^*a,b,d*^	**<0.001** (0.312)
PP (mmHg)	6713	69±14^*a*^	77 ± 15^*a,b*^	82 ± 15^*a,b,c*^	86 ± 16^*a,b,c,d*^	**<0.001** (0.199)
MCAv (cm/s)	5511	59±13^*a*^	60±13^*a*^	58±14^*a*^	56 ± 13^*b,c,d*^	**<0.001** (0.021)
Cerebral PI (A.U.)	0.80±0.09^*a*^	0.76±0.09^*a*^	0.75±0.09^*a*^	0.75±0.09^*a*^	0.83 ± 0.10^*a,b,c,d*^	**<0.001** (0.096)
CVRi (mmHg/cm/s)	1.9 ± 0.48	1.9 ± 0.49	2.1 ± 0.51^*a,b*^	2.4 ± 0.60^*a,b,c*^	2.4 ± 0.60^*a,b,c*^	**<0.001** (0.112)
ETCO_2_ (mmHg)	393	39 ± 4	38 ± 4	37 ± 4*^b,c^*	38 ± 3*^a,b,c^*	**<0.001** (0.021)

*Values are expressed as means ± SD. Data for isometric handgrip exercise are divided into tertiles (Exercise T1, T2, and T3). Data for post-exercise ischemia (PEI) presented during the final 60s of PEI. Cerebral PI, cerebral pulsatility index; CO, cardiac output; CVRi, cerebrovascular resistance index; DBP, diastolic blood pressure; ETCO2, end-tidal CO2; HR, heart rate; MAP, mean arterial blood pressure; MCAv, middle cerebral artery blood velocity; PP, pulse pressure; SBP, systolic blood pressure. Bolded P-values indicate a significant effect of condition. **^a^** P < 0.05 vs. baseline, **^b^** P < 0.05 vs. exercise T1, **^c^** P < 0.05 vs. exercise T2, **^d^** P < 0.05 vs. exercise T3; (n = 68; repeated measures ANOVA with Bonferroni post hoc testing. Effect sizes calculated as eta squared).*

**TABLE 3:** Percent change from baseline in cardiovascular and cerebrovascular variables during isometric handgrip exercise and post-exercise ischemia.

Variable	Exercise T1	Exercise T2	Exercise T3	PEI	*P*-value (effect size)
HR (%)	10 ± 6	17±9^*b*^	25 ± 12^*b,c*^	8 ± 7^*c,d*^	**<0.001** (0.348)
MAP (%)	7 ± 5	19±9^*b*^	27 ± 11^*b,c*^	22 ± 9^*b,d*^	**<0.001** (0.432)
SBP (%)	5 ± 5	17±8^*b*^	25 ± 10^*b,c*^	23 ± 9^*b,c*^	**<0.001** (0.465)
DBP (%)	7 ± 6	19±10^*b*^	26 ± 12^*b,c*^	18 ± 9^*b,d*^	**<0.001** (0.356)
PP (%)	3 ± 6	12±8^*b*^	7 ± 5^*b,c*^	29 ± 14^*b,c,d*^	**<0.001** (0.555)
MCAv (%)	7 ± 6	8 ± 7	5±10^*c*^	0 ± 10^*b,c,d*^	**<0.001** (0.115)
Cerebral PI (%)	−5 ± 4	−6 ± 6	−5 ± 7	4 ± 7^*b,c,d*^	**<0.001** (0.312)
CVRi (%)	0 ± 6	11±9^*b*^	22 ± 17^*b,c*^	23 ± 16^*b,c*^	**<0.001** (0.354)

*Values are expressed as means ± SD. Data for isometric handgrip exercise are divided into tertiles (Exercise T1, T2, and T3). Data for post-exercise ischemia (PEI) presented during the final 60s of PEI. Cerebral PI, cerebral pulsatility index; CVRi, cerebrovascular resistance index; DBP, diastolic blood pressure; HR, heart rate; MAP, mean arterial blood pressure; MCAv, middle cerebral artery blood velocity; PP, pulse pressure; SBP, systolic blood pressure. Bolded P-values indicate a significant effect of condition. **^b^**P < 0.05 vs. exercise T1, **^c^**P < 0.05 vs. exercise T2, **^d^**P < 0.05 vs. exercise T3; (n = 68; repeated measures ANOVA with Bonferroni post hoc testing. Effect sizes calculated as eta squared).*

### Associations Between Cardiovascular and Cerebrovascular Responses to Sympathoexcitatory Stimuli and White Matter Hyperintensities

**FIGURE 4:**
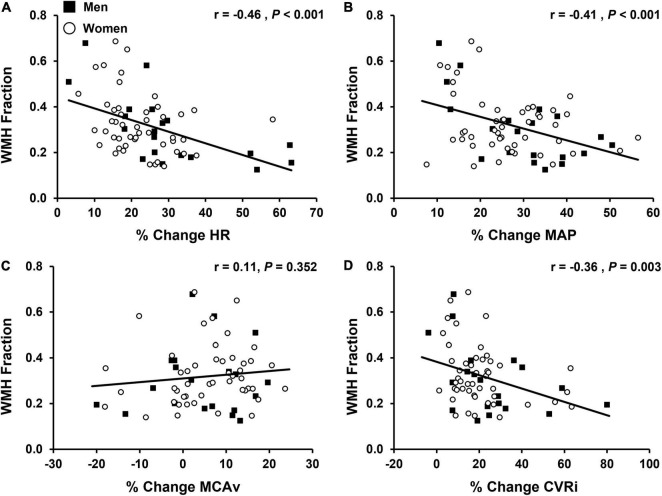
Associations between the percent change (% change) from baseline values in cardiovascular and cerebrovascular variables during the final tertile of isometric handgrip exercise (EX T3) and white matter hyperintensity (WMH) fraction (n = 68). WMH fraction was calculated by dividing WMH lesion volume by intracranial volume, converting to a percentage, and applying a cubic root transformation to reduce skewness. Men are shown in black squares and women are shown in open circles. **(A)** % change in heart rate, HR vs. WMH; **(B)** % change in mean arterial pressure, MAP vs. WMH; **(C)** % change in middle cerebral artery blood velocity, MCAv vs. WMH; **(D)** % change incerebrovascular resistance index, CVRi vs. WMH. Note: data and statistics shown in figure have not been adjusted for age at MRI, sex, APOE 4 status, or work performed during isometric handgrip exercise. After adjustment for confounding variables, there was a negative association between the % change in HR (*P* = 0.002), MAP (*P* = 0.002), and CVRi (*P* = 0.045) during the final tertile of isometric handgrip exercise and WMH fraction. Corresponding results from multiple linear regression analyses are presented in Tables 4, 5.

All multiple linear regressions between cardiovascular and cerebrovascular variables at rest and WMH fraction were adjusted for age at MRI, sex, and *APOE* ε4 status. HR (*P* = 0.006), MAP (*P* < 0.001), SBP (*P* = 0.002), DBP (*P* < 0.001), and PP (*P* < 0.001) at rest were positively associated with WMH fraction. There were no significant associations between the cerebrovascular variables at rest (MCAv, cerebral PI, or CVRi) and WMH fraction (*P* > 0.05 for all). All multiple linear regressions between cardiovascular and cerebrovascular responses to sympathoexcitatory stimuli and WMH fraction were adjusted for age at MRI, sex, *APOE* ε4 status, and work performed during the IHG exercise protocol. Results of multiple linear regression analyses during the final tertile of IHG exercise and WMH fraction are presented in Table 4. There was a negative association between the percent change in HR (*P* = 0.002), MAP (*P* = 0.002), SBP (*P* = 0.005), DBP (*P* = 0.002), and CVRi (*P* = 0.045) during the final tertile of IHG exercise and WMH fraction (Table 4). Unadjusted individual data between the percent change in HR, MAP, MCAv, and CVRi during the final tertile of IHG exercise and WMH fraction are presented in Figure 4. There were no significant associations between cardiovascular and cerebrovascular variables during post-exercise ischemia and WMH fraction (Table 5).

**TABLE 4:** Results of multiple linear regression analysis between cardiovascular and cerebrovascular variables during isometric handgrip exercise and white matter hyperintensity fraction.

Variable	Standardized β	*P*-value
**HR**		
HR (bpm)	0.067	0.586
% Change HR	–0.432	**0.002**
**MAP**		
MAP (mmHg)	–0.018	0.886
% Change MAP	–0.380	**0.002**
**SBP**		
SBP (mmHg)	0.031	0.806
% Change SBP	–0.361	**0.005**
**DBP**		
DBP (mmHg)	–0.094	0.479
% Change DBP	–0.383	**0.002**
**PP**		
PP (mmHg)	0.119	0.348
% Change PP	–0.110	0.406
**MCAv**		
MCAv (cm/s)	–0.022	0.868
% Change MCAv	–0.005	0.971
**Cerebral** **PI**		
PI (A.U.)	0.127	0.350
% Change PI	0.181	0.145
**CVRi**		
CVRi (mmHg/cm/s)	0.005	0.973
% Change CVRi	–0.271	**0.045**

*Data presented as standardized β estimates for raw values and the percent change (% change) from baseline during the final tertile of isometric handgrip exercise. Cerebral PI, cerebral pulsatility index; CVRi, cerebrovascular resistance index; DBP, diastolic blood pressure; HR, heart rate; MAP, mean arterial blood pressure; MCAv, middle cerebral artery blood velocity; PP, pulse pressure; SBP, systolic blood pressure. White matter hyperintensity (WMH) fraction was calculated by dividing WMH lesion volume by intracranial volume, converting to a percentage, and applying a cubic root transformation to reduce skewness. Linear regression estimates adjusted for age at MRI (y), sex (Women = 1; Men = 0), APOE ε4 status (APOE ε4 positive = 1; APOE ε4 negative = 0), and work performed during the isometric handgrip exercise protocol (kg/s). (n = 68. Bolded p-values indicate a significant association between the cardiovascular or cerebrovascular variable and WMH fraction).*

**TABLE 5:** Results of multiple linear regression analysis between cardiovascular and cerebrovascular variables during post-exercise ischemia and white matter hyperintensity fraction.

Variable	Standardized β	*P*-value
**HR**		
HR (bpm)	0.232	0.059
% Change HR	–0.135	0.265
**MAP**		
MAP (mmHg)	0.180	0.153
% Change MAP	–0.143	0.256
**SBP**		
SBP (mmHg)	0.181	0.149
% Change SBP	–0.146	0.251
**DBP**		
DBP (mmHg)	0.141	0.270
% Change DBP	–0.149	0.231
**PP**		
PP (mmHg)	0.171	0.170
% Change PP	–0.105	0.408
**MCAv**		
MCAv (cm/s)	0.034	0.799
% Change MCAv	0.116	0.356
**Cerebral** **PI**		
PI (A.U.)	–0.037	0.775
% Change PI	–0.037	0.759
**CVRi**		
CVRi (mmHg/cm/s)	0.061	0.647
% Change CVRi	–0.183	0.162

*Data presented as standardized β estimates for raw values and the percent change (% change) from baseline during the finals 60s of post-exercise ischemia. Cerebral PI, cerebral pulsatility index; CVRi, cerebrovascular resistance index; DBP, diastolic blood pressure; HR, heart rate; MAP, mean arterial blood pressure; MCAv, middle cerebral artery blood velocity; PP, pulse pressure; SBP, systolic blood pressure. White matter hyperintensity (WMH) fraction was calculated by dividing WMH lesion volume by intracranial volume, converting to a percentage, and applying a cubic root transformation to reduce skewness. Linear regression estimates adjusted for age at MRI (y), sex (Women = 1; Men = 0), APOE ε4 status (APOE ε4 positive = 1; APOE ε4 negative = 0), and work performed during the isometric handgrip exercise protocol (kg/s). (n = 68).*

Additionally, we evaluated the effect of controlled hypertension on our findings. After correction for age at MRI, sex, *APOE* ε4 status, and controlled hypertension, HR (*P* = 0.023), MAP (*P* = 0.006), SBP (*P* = 0.012), and DBP (*P* = 0.008) at rest remained positively associated with WMH fraction. There were no significant associations between PP, MCAv, cerebral PI, or CVRi at rest and WMH fraction after correction for confounding variables (*P* > 0.05 for all). The results of multiple linear regression analyses corrected for age at MRI, sex, *APOE* ε4 status, work performed during the IHG exercise protocol, and controlled hypertension during IHG exercise and post-exercise ischemia are located in Supplementary Tables 1, 2. Following correction for controlled hypertension, the inverse associations between the percent change in HR (*P* = 0.014), MAP (*P* = 0.013), SBP (*P* = 0.032), and DBP (*P* = 0.008) during the final tertile of IHG exercise and WMH fraction remained significant (Supplementary Table 1). However, there was no longer a negative association between the percent change in CVRi during the final tertile of IHG exercise and WMH fraction (*P* = 0.136, Supplementary Table 1). There were no significant associations between cardiovascular and cerebrovascular variables during post-exercise ischemia and WMH fraction (Supplementary Table 2).

## Discussion

The primary aim of this study was to evaluate associations between cardiovascular and cerebrovascular responses to sympathoexcitatory stimuli and WMH burden. The novel finding of this study was that a lower percent change in HR, BP, and CVRi in response to IHG exercise was associated with greater WMH burden in middle-aged and older adults, which was opposite of our original hypothesis. These results persisted when accounting for potentially confounding variables including age at MRI, sex, *APOE*ε4 status, and work performed during the IHG exercise protocol. Our results indicate that individuals with greater WMH burden may have impaired cardiovascular and cerebrovascular responses to sympathoexcitatory stress. Together, our findings suggest that blunted HR, BP and CVRi in response to acute sympathoexcitatory stress are associated with greater WMH burden in cognitively unimpaired middle-aged and older adults.

We have previously reported a 10-14% increase in HR, 7-20% increase in MAP, and 9-13% increase in MCAv in response to IHG exercise at 30% of MVC until fatigue in postmenopausal women with or without a history of hypertensive pregnancy (Ranadive et al., 2017; Miller et al., 2019). Consistent with our previous work and others, we observed similar sustained increases in cardiovascular (Lewis et al., 1983; Sander et al., 2010; Ranadive et al., 2017; Lee et al., 2021) and cerebrovascular variables (Miller et al., 2019) during IHG exercise and post-exercise ischemia. A unique aspect of this study is the inclusion of cerebral hemodynamics (MCAv and CVRi) to assist in interpretation of hemodynamic changes in the cerebral vessels in response to two sympathoexcitatory stressors. In response to IHG exercise at 40% MVC, we observed a 25% increase in HR, a 27% increase in MAP, a 5% increase in MCAv, and a 22% increase in CVRi during the final tertile of IHG exercise. Compared to our previous work, the larger increases in HR, MAP, SBP, and DBP observed in this study are likely due to the inclusion of both men and women as well as a greater relative workload (40 vs. 30% MVC). Our findings build upon our previous work in this area by including a larger sample size of both sexes and evaluating associations between cardiovascular and cerebrovascular responses to acute sympathoexcitatory stimuli and WMH. To our knowledge, this is the first study to examine associations between cardiovascular and cerebrovascular responses to sympathoexcitatory stimuli and WMH burden. All participants in this study were enrolled in Wisconsin ADRC cohorts. Participants enrolled in these cohorts are part of a larger longitudinal study of over 1,200 individuals focused on early detection of AD, identification of both protective and risk factors, and developing strategies to delay onset and progression of AD (Johnson et al., 2017). The participants recruited for this study were relatively young and were healthy, free of cardiovascular disease (other than controlled hypertension *n* = 13), dementia of any kind, and history of clinically significant stroke, cerebrovascular disease, or other major neurological disorders. Therefore, collecting physiological data in this cohort provides insight beyond aging alone. As a result, the stressors used in this study in cognitively unimpaired middle-aged and older adults have the potential to serve as useful tools for early detection and identification of risk factors associated with cognitive decline and dementia.

The relationship between elevated BP at rest and WMH burden has been previously reported (van Dijk et al., 2004) and elevated BP and BP variability during the middle-aged years are associated with increased WMH burden (Dufouil et al., 2001; Havlik et al., 2002; Wartolowska and Webb, 2021). Elevated BP at midlife is also associated with increased risk of cognitive decline (Swan et al., 1998; DeCarli et al., 2001), dementia (McGrath et al., 2017; Walker et al., 2019), and AD (Lennon et al., 2019; Ou et al., 2020). However, epidemiological and longitudinal studies only provide insight into associations between BP values at rest and WMH burden or retrospective analysis based on historical BP measurements. Cardiovascular and cerebrovascular responses to acute or chronic physiological stimuli in healthy adults may reveal dysfunction in the systemic and cerebral circulation prior to significant damage to or changes in brain volumes. Indeed, greater BP responses to mental stress are associated with poor performance on cognitive challenges (Waldstein and Katzel, 2005) and WMH burden (Waldstein et al., 2004). Acceleration of WMH burden occurs prior to presentation of MCI (Silbert et al., 2012) and midlife represents a unique period during the presymptomatic phase of dementia in which intervention may be beneficial to delay the onset of cognitive decline. As such, identifying potential mechanisms contributing to the increase in WMH burden during midlife may be important for understanding the progression from normal cognition to presentation of symptoms of cognitive decline.

Isometric handgrip exercise followed by a period of post-exercise ischemia represent acute physiological stimuli that elicit a marked increase in HR, BP, and sympathetic nervous system activity. In agreement with our original hypothesis, the expected response to a sympathoexcitatory stimulus is a substantial increase in HR, BP, and CVRi. Indeed, we observed a 25% increase in HR, 27% increase in MAP, 25% increase in SBP, 26% increase in DBP, and a 22% increase in CVRi during the final tertile of IHG exercise. Moreover, each of these variables remained elevated during post-exercise ischemia. Contrary to our hypothesis, however, a lower percent change in HR, MAP, SBP, DBP, and CVRi in response to IHG exercise was associated with greater WMH burden in middle-aged and older adults. One explanation for these findings is that failure to increase resistance in the cerebral circulation (i.e., CVRi) may lead to propagation of highly pulsatile blood flow into the delicate microcirculation suggesting that individuals with greater WMH burden may have impaired regulation of cerebral blood flow. Along these lines, following an acute hypertensive stimulus (a single bout of resistance training), older adults demonstrated a greater increase in cerebral PI despite no change in mean MCAv compared with young adults (Rosenberg et al., 2020). These results suggest that cerebral blood flow regulation may be impaired following an acute hypertensive stimulus in older adults, which may lead to greater transmission of pulsatile flow into the microcirculation and increased risk of end-organ damage.

Consistent with this idea, carotid artery PP, carotid PI, cerebral PI, and aortic stiffness are associated with increased risk for silent subcortical infarcts (Mitchell et al., 2011). Additionally, aortic stiffness is associated with higher WMH volume and carotid PI is associated with lower gray and white matter volumes (Mitchell et al., 2011). In the cerebral circulation, cerebral PI is positively associated with greater WMH volume in middle-aged and older adults (Tarumi et al., 2014). These findings suggest that aortic stiffness and transmission of pulsatile flow into the microcirculation may lead to quantifiable changes in brain volume and increased WMH burden. A recent study conducted by Tarumi et al. in young adults investigated cardiovascular variables and cerebral blood flow in response to repeated bouts of rhythmic handgrip exercise using phase-contrast MRI (Tarumi et al., 2021). Using a similar relative intensity of handgrip exercise (30-40% MVC), HR, BP, CVRi, cerebral blood flow, and respiratory rate increased during rhythmic handgrip exercise despite no change in vessel cross-sectional area (Tarumi et al., 2021). Thus, higher resistance in the cerebral vessels may attenuate an increase in cerebral blood flow and prevent cerebral hyperperfusion during rhythmic handgrip exercise, which may represent a compensatory myogenic response to a hypertensive stimulus in order to dampen pulsatile flow. Failure to increase resistance in the cerebral vessels (indicated by a smaller increase in CVRi) during an acute sympathoexcitatory stimulus suggests a potential lack of active vasoconstriction occurring in the cerebral circulation that may allow for propagation of pulsatile flow into the microcirculation. Broadly, acute instances of physiological stressors that affect BP and cerebral blood flow, experienced across the lifespan, may affect white matter health. We report that individuals who demonstrated greater increases in HR, BP, and CVRi in response to IHG exercise had lower WMH burden, which was opposite of our hypothesis. It is possible that increases in CVRi during a sympathoexcitatory stress, indicating active vasoconstriction, may protect the cerebral circulation from increases in pulsatile flow. For example, in spontaneously hypertensive rats, smooth muscle hypertrophy in the cerebral arteries occurs, leading to increased cerebrovascular resistance (Tayebati et al., 2012). Changes in structure and function in the cerebral circulation may protect the brain from high perfusion pressures during periods of elevated BP and augmented cerebral blood flow (Faraci and Heistad, 1990; Tayebati et al., 2012). Alternatively, individuals with greater WMH burden or reduced white matter integrity (which was not measured in the present study) may have impaired cardiovascular responses to sympathoexcitatory stress. However, this is unlikely as WMH volumes in the present study are low, likely due to the average age of participants in this study being only ∼63 years.

In agreement with the findings from Tarumi et al., we report significant increases in HR, BP, MCAv, and CVRi during IHG exercise in middle-aged and older adults. Yet, the cardiovascular and cerebrovascular responses were highly variable. While IHG exercise was performed at the same relative intensity for all participants, we observed a wide range of responses in HR (3-63% increase), MAP (8-57% increase), MCAv (−20 to + 23% change), and CVRi (−4 to + 80% change) during the final tertile of IHG exercise. In agreement with our findings, the percent change in MAP and MCAv in response to rhythmic handgrip exercise appears to be heterogeneous (Giller et al., 2000) whereby MCAv may decrease in some individuals. Our findings indicate that middle-aged and older adults demonstrate varying responses to an acute sympathoexcitatory stimulus performed at the same relative intensity. IHG exercise represents an acute sympathoexcitatory stimulus that may be comparable to carrying an object for an extended period of time. As these types of activities are performed frequently throughout activities of daily living, evaluating the cardiovascular and cerebrovascular response to repeated bouts of acute sympathoexcitatory stress may be important for understanding changes in the brain prior to presentation of symptoms of cognitive decline. Altered expected responses to acute physiological stressors such as IHG exercise, which induce brief, large increases in BP, may be associated with measurable changes in brain volume and WMH.

Hypertension is associated with increased risk of developing WMH (Dufouil et al., 2001). Although we may be underpowered to adequately control for controlled hypertension (*n* = 13, 19% of participants) in our population of middle-aged and older adults, associations with WMH fraction were evaluated with controlled hypertension included in the linear model (in addition to age at MRI, sex, *APOE* ε4 status, and work performed during the IHG exercise protocol). After adjusting for controlled hypertension, negative associations between the percent change in HR, MAP, SBP, and DBP during IHG exercise and WMH fraction remained significant. Yet, there was no longer an association between the percent change in CVRi during IHG exercise and WMH fraction. Although the individuals with controlled hypertension were evenly distributed throughout the entire group for the variables of interest, previous work has suggested that older hypertensive adults demonstrate greater increases in raw MAP values and sympathetic nerve activity in response to IHG exercise performed at 30 and 40% MVC compared with normotensive older adults (Delaney et al., 2010). Additionally, older hypertensive adults exhibit a more rapid increase in MAP and sympathetic nerve activity (within the first 10s of muscular contraction) compared with normotensive older adults (Greaney et al., 2015). In the aforementioned studies, older adults in the hypertensive group currently taking antihypertensive medication (80% of participants) were instructed to refrain from taking medication for two days prior to the experimental study (Delaney et al., 2010; Greaney et al., 2015). In the present study, there were no differences in the change in raw MAP and CVRi values between adults with controlled hypertension and those who were normotensive (*P* > 0.05 for both), although the aims of the present study did not include evaluating the effects of controlled hypertension. One possible explanation for the discrepancy between previous studies (Delaney et al., 2010; Greaney et al., 2015) and our findings is that individuals with uncontrolled hypertension were excluded from our study. Additionally, participants in the present study were instructed to take prescription medication on the day of the study, which could have influenced the response to IHG and post-exercise ischemia. Nevertheless, when controlled hypertension was added to the linear model, the inverse association between the percent change in HR, MAP, SBP, and DBP during IHG exercise and WMH fraction remained significant (*P* < 0.05 for all; Supplementary Table 1). These results suggest that regardless of controlled hypertension status, an attenuated increase in HR and BP in response to IHG exercise is associated with greater WMH burden in middle-aged and older adults.

## Limitations

In order to determine CVRi, we measured the cerebral hemodynamic response to IHG exercise in the MCA using TCD. A major assumption of TCD is that the MCA diameter remains constant at rest and during a stimulus. Previous work evaluating MCAv and MCA diameter in response to rhythmic handgrip exercise has demonstrated that MCA diameter may decrease by ∼ 2% in adults aged 20-59 years old suggesting active vasoconstriction in the cerebral circulation during rhythmic handgrip exercise (Verbree et al., 2017). However, the cross sectional area of the internal carotid and vertebral arteries do not change in response to rhythmic handgrip exercise performed at 30-40% MVC in young adults (Tarumi et al., 2021). It is possible that IHG exercise causes vasoconstriction of the MCA which may cause an increase in MCAv despite little or no change in cerebral blood flow (Giller et al., 2000), though the effect of isometric versus rhythmic handgrip exercise on MCA diameter is currently unknown. While the present study evaluated MCAv in response to IHG exercise in a robust sample size of middle-aged and older adults, the cerebrovascular response to IHG exercise and potential associations with WMH burden should be interpreted with caution. Additionally, all participants performed the IHG exercise protocol using their left hand, regardless of handedness. Due to the experimental setup, we evaluated MCAv in the left (ipsilateral) MCA. When MCAv was evaluated in both the right and left MCA during rhythmic handgrip exercise in adults aged 21-43 years old, previous studies have shown that MCAv increased in the contralateral side only (Jørgensen et al., 1993; Linkis et al., 1995). In the present study, we report a significant, albeit variable, increase in MCAv in the ipsilateral MCA during IHG exercise in adults aged 55-69 years old, which we interpret as a global response. It is possible that we could have observed a greater increase in MCAv in the contralateral (right) MCA during IHG exercise, and follow-up studies should perform bilateral MCA assessments. Lastly, arterial CO_2_ is a powerful regulator of cerebral vascular tone (Brian, 1998) and we utilized ETCO_2_ as a surrogate measure of arterial CO_2_. A reduction in ETCO_2_ may cause cerebral vasoconstriction and potentially a decrease in cerebral blood flow. We observed a small (1-2 mmHg), but significant, reduction in ETCO_2_ during the final tertile of IHG exercise which persisted during post-exercise ischemia. Similarly, reductions in ETCO_2_ have also been observed during isometric (Muza et al., 1983; Fontana et al., 1993), and rhythmic handgrip exercise (Jørgensen et al., 1993; Tarumi et al., 2021). It is possible that a 1-2 mmHg decrease in ETCO_2_ could have resulted in a decrease in MCAv in this study.

## Conclusion

In summary, IHG exercise performed at 40% MVC until fatigue elicited significant increases in HR, BP, MCAv, and CVRi in cognitively unimpaired middle-aged and older adults. Individuals with greater WMH burden demonstrated a lower percent change in HR, BP, and CVRi in response to IHG exercise. Together, these results indicate an inverse association between WMH burden and cardiovascular and cerebrovascular responses to a sympathoexcitatory stimulus.

## Data Availability Statement

The raw data supporting the conclusions of this article will be made available by the authors, without undue reservation.

## Ethics Statement

The studies involving human participants were reviewed and approved by University of Wisconsin-Madison Institutional Review Board. The patients/participants provided their written informed consent to participate in this study.

## Author Contributions

JB conceived and designed the research. AP, KM, AC, NE, AH, and JB performed the experiments. AP analyzed the data. AP, KC, and JB interpreted the results of experiments. AP prepared the figures. AP and JB drafted the manuscript. All authors edited and revised the manuscript, and approved the final version of the manuscript.

## Conflict of Interest

The authors declare that the research was conducted in the absence of any commercial or financial relationships that could be construed as a potential conflict of interest.

## Publisher’s Note

All claims expressed in this article are solely those of the authors and do not necessarily represent those of their affiliated organizations, or those of the publisher, the editors and the reviewers. Any product that may be evaluated in this article, or claim that may be made by its manufacturer, is not guaranteed or endorsed by the publisher.
