# Supplementary material for: Sympathoexcitatory Responses to Isometric Handgrip Exercise Are Associated With White Matter Hyperintensities in Middle-Aged and Older Adults
Source: Front Aging Neurosci. 2022 Jul 11;14:888470. doi: 10.3389/fnagi.2022.888470 (PMC9309556; doi:10.3389/fnagi.2022.888470)
Supplement: Supplementary file 1 [file Data_Sheet_1.docx]

| **Variable** | **Standardized β** | ***P*-value** |
| --- | --- | --- |
| **HR**  HR (bpm)  % Change HR  **MAP**  MAP (mmHg)  % Change MAP  **SBP**  SBP (mmHg)  % Change SBP  **DBP**  DBP (mmHg)  % Change DBP  **PP**  PP (mmHg)  % Change PP  **MCAv**  MCAv (cm/s)  % Change MCAv  **Cerebral** **PI**  PI (au)  % Change PI  **CVRi**  CVRi (mmHg/cm/s)  % Change CVRi | 0.044  -0.357  -0.002  -0.316  0.030  -0.286  -0.063  -0.327  0.094  -0.051  0.026  -0.014  0.132  0.134  -0.045  -0.202 | 0.709  **0.014**  0.986  **0.013**  0.805  **0.032**  0.624  **0.008**  0.445  0.693  0.843  0.907  0.312  0.271  0.737  0.136 |

**Supplementary Table 1.** Results of multiple linear regression analysis between cardiovascular and cerebrovascular variables during isometric handgrip exercise and white matter hyperintensity fraction additionally adjusted for controlled hypertension.

Data presented as standardized β estimates for raw values and the percent change (% change) from baseline during the final tertile of isometric handgrip exercise. Cerebral PI, cerebral pulsatility index; CVRi, cerebrovascular resistance index; DBP, diastolic blood pressure; HR, heart rate; MAP, mean arterial blood pressure; MCAv, middle cerebral artery blood velocity; PP, pulse pressure; SBP, systolic blood pressure. White matter hyperintensity (WMH) fraction was calculated by dividing WMH lesion volume by intracranial volume, converting to a percentage, and applying a cubic root transformation to reduce skewness. Linear regression estimates adjusted for age at MRI (y), sex (Women = 1; Men = 0), *APOE* ε4 status (*APOE* ε4 positive = 1; *APOE* ε4 negative = 0), controlled hypertension (controlled hypertension = 1; No controlled hypertension = 0), and work performed during the isometric handgrip exercise protocol (kg/s). n = 68.

| **Variable** | **Standardized β** | ***P*-value** |
| --- | --- | --- |
| **HR**  HR (bpm)  % Change HR  **MAP**  MAP (mmHg)  % Change MAP  **SBP**  SBP (mmHg)  % Change SBP  **DBP**  DBP (mmHg)  % Change DBP  **PP**  PP (mmHg)  % Change PP  **MCAv**  MCAv (cm/s)  % Change MCAv  **Cerebral** **PI**  PI (au)  % Change PI  **CVRi**  CVRi (mmHg/cm/s)  % Change CVRi | 0.155  -0.147  0.140  -0.132  0.143  -0.102  0.106  -0.153  0.138  -0.031  0.074  0.084  0.007  -0.027  -0.004  -0.145 | 0.217  0.207  0.252  0.277  0.239  0.412  0.394  0.203  0.252  0.809  0.566  0.490  0.958  0.816  0.975  0.257 |

**Supplementary Table 2.** Results of multiple linear regression analysis between cardiovascular and cerebrovascular variables during post-exercise ischemia and white matter hyperintensity fraction additionally adjusted for controlled hypertension.

Data presented as standardized β estimates for raw values and the percent change (% change) from baseline during the finals 60s of post-exercise ischemia. Cerebral PI, cerebral pulsatility index; CVRi, cerebrovascular resistance index; DBP, diastolic blood pressure; HR, heart rate; MAP, mean arterial blood pressure; MCAv, middle cerebral artery blood velocity; PP, pulse pressure; SBP, systolic blood pressure. White matter hyperintensity (WMH) fraction was calculated by dividing WMH lesion volume by intracranial volume, converting to a percentage, and applying a cubic root transformation to reduce skewness. Linear regression estimates adjusted for age at MRI (y), sex (Women = 1; Men = 0), *APOE* ε4 status (*APOE* ε4 positive = 1; *APOE* ε4 negative = 0), controlled hypertension (controlled hypertension = 1; No controlled hypertension = 0), and work performed during the isometric handgrip exercise protocol (kg/s). n = 68.
